# Acute Kidney Injury and Electrolyte Disorders Following Chimeric Antigen Receptor T-cell (CAR T-cell) Therapy in Adults With Hematologic Malignancies: A Retrospective Study

**DOI:** 10.7759/cureus.101460

**Published:** 2026-01-13

**Authors:** Omar A AlShammari, Mohammad Alwadi, Ibrahim Abuqurayn, Hassan Alshehri, Ebtesam AlMadi, Bilal AlBtoosh, Khalid I AlMatham, Fadel Alrowaie

**Affiliations:** 1 Internal Medicine, King Fahad Medical City, Riyadh, SAU; 2 Internal Medicine, Alfaisal University College of Medicine, Riyadh, SAU; 3 Hematology, King Fahad Medical City, Riyadh, SAU; 4 Hematology and Medical Oncology, King Fahad Medical City, Riyadh, SAU

**Keywords:** acute kidney injury, car-t therapy, electrolyte disorders, hematologic malignancies, nephrotoxicity

## Abstract

Introduction

Renal complications are increasingly recognized after chimeric antigen receptor (CAR) T-cell therapy. We evaluated the incidence and severity of acute kidney injury (AKI), the prevalence of electrolyte disturbances, and clinical factors associated with AKI in adult patients with hematological malignancies.

Methods

We retrospectively reviewed all adult patients with hematological malignancies who received CAR T-cell therapy at a single tertiary center between November 2023 and April 2025. Baseline demographics, comorbidities, prior therapies, and post-infusion events were collected from electronic medical records. AKI was defined and staged according to the Kidney Disease: Improving Global Outcomes (KDIGO) guidelines based on serum creatinine (Cr) levels. Electrolyte disturbances (sodium, potassium, and phosphate) occurring within 100 days post-infusion were recorded. Data were analyzed using R statistical software (version 4.5.1). Continuous variables are presented as the mean ± SD or median (IQR). Groups were compared using t-tests, Wilcoxon rank-sum tests, and Fisher’s exact tests. Associations between AKI and patient characteristics were assessed using logistic regression. Hospitalization outcomes were evaluated using Kaplan-Meier and Cox proportional hazards models. A p-value < 0.05 was considered statistically significant.

Results

This study included 16 patients (mean age: 56.7 ± 17.7 years; 50% male), 25% of whom developed AKI (all stage 1). Pre-existing chronic kidney disease (CKD), baseline Cr ≥75 μmol/L, and diabetes mellitus showed the strongest associations with AKI. Electrolyte disturbances were common, particularly potassium and phosphate abnormalities. AKI was not significantly associated with ICU admission, longer hospitalization, or early mortality.

Conclusion

In this cohort, renal events after CAR T-cell therapy were mainly mild. Pre-existing CKD and diabetes mellitus were associated with an increased risk of AKI. Therefore, we suggest close monitoring of kidney function and other risk factors (such as nephrotoxic medications, contrast exposure, and dehydration) during treatment. Further studies with larger cohorts and longer follow-up are required to clarify the mechanisms of renal impairment and better assess outcomes.

## Introduction

Chimeric antigen receptor (CAR)-T-cell therapy is a promising new approach for treating hematological malignancies. The first CAR-T-cell therapy was approved by the US FDA in 2017 for treating children and young adults with acute lymphoblastic leukemia (ALL). CAR-T cells are genetically modified immune cells engineered to identify and bind specific antigens expressed on malignant cells. The therapeutic process begins with the collection of autologous T lymphocytes, followed by ex vivo genetic modification, expansion, and activation before reinfusion into the patient. This genetic engineering enables surface expression of CARs, allowing antigen-specific recognition of tumor cells and subsequent T-cell activation, resulting in targeted cytotoxicity against cancer cells [1,2].

Current indications for CAR-T-cell therapy have expanded to include additional hematological malignancies, such as relapsed or refractory B-cell leukemia, lymphoma, and multiple myeloma, with evidence suggesting disease remission and improvement in overall outcomes [3-12].

The indications for CAR-T-cell therapy continue to evolve, and growing evidence supports its benefits beyond its initial indications, including for non-neoplastic conditions such as autoimmune disorders (e.g., severe refractory systemic lupus erythematosus (SLE)), myocardial fibrosis, and infectious diseases such as AIDS [13].

Although clinically effective, CAR-T-cell therapy has been linked to multiple treatment-related toxicities, notably immune effector cell-associated neurotoxicity syndrome (ICANS), cytokine release syndrome (CRS), CAR-T-cell therapy-associated haemophagocytic lymphohistiocytosis (carHLH), cardiac toxicity, and acute kidney injury (AKI) [14].

Renal toxicity, including AKI, has a broad incidence rate, affecting 5%-33% of patients with non-Hodgkin lymphoma (NHL) undergoing CAR-T-cell therapy via different mechanisms (pre-renal, renal, and post-renal) [15,16]. Patients undergoing CAR-T-cell therapy are exposed to several factors that may predispose them to AKI, including underlying malignancy, volume depletion, exposure to combination anticancer therapies with nephrotoxic potential, malignancy-related urinary tract obstruction, and systemic inflammatory states such as CRS that can impair renal perfusion and hemodynamic stability [17].

Furthermore, Gupta S et al. reported a high incidence of electrolyte disorders after CAR-T-cell infusion, including hyponatremia (75%), hypokalemia (56%), and hypophosphatemia (51%). However, the underlying causes remain unclear [17-19].

Accordingly, this study aimed to evaluate the incidence of AKI and the frequency of electrolyte abnormalities, identify relevant clinical predictors, and examine the influence of baseline patient characteristics on the risk of AKI among adults with hematologic malignancies receiving CAR-T-cell therapy. In addition, the study aimed to assess the association between AKI and clinical outcomes, including early mortality and length of hospitalization.

## Materials and methods

Study design and data collection

A retrospective cohort study was conducted involving adult patients with hematologic malignancies who received CAR-T-cell therapy at our institution between November 2023 and April 2025. All individuals treated with CAR-T-cell therapy during the study period were included, and no exclusion criteria were applied.

Baseline patient information was obtained through review of electronic medical records and included demographic variables such as age and sex, as well as pre-existing comorbid conditions, including hypertension, diabetes mellitus, and chronic kidney disease (CKD). Clinical data related to CAR-T-cell therapy were collected, including treatment indication, number of prior therapies, history of autologous or allogeneic hematopoietic cell transplantation, and lymphodepleting chemotherapy regimens. Post-treatment clinical events were also assessed, including ICU admission and exposure to potentially nephrotoxic medications such as vancomycin, nonsteroidal anti-inflammatory drugs, acyclovir, and trimethoprim-sulfamethoxazole. Medical records were reviewed to identify the occurrence of cytokine release syndrome, immune effector cell-associated neurotoxicity syndrome, and tumor lysis syndrome. Clinical outcomes, including hospitalization duration and its relationship with AKI severity, were evaluated.

Renal function was assessed using serum creatinine concentrations and estimated glomerular filtration rate, calculated using the Chronic Kidney Disease Epidemiology Collaboration equation. For patients who developed AKI, serum creatinine levels were followed for up to 30 days to evaluate renal recovery.

AKI was classified according to the Kidney Disease: Improving Global Outcomes (KDIGO) criteria into stages 1 through 3. Stage 1 was defined as an increase in serum creatinine of at least 0.3 mg/dL (26.5 μmol/L) within 48 hours or a 1.5-1.9-fold increase from baseline within seven days. Stage 2 was defined as a 2.0-2.9-fold increase from baseline, while stage 3 was defined as a threefold or greater increase from baseline, an absolute serum creatinine level of at least 4.0 mg/dL (353.6 μmol/L), or the initiation of renal replacement therapy. Urine output criteria were not included because of the retrospective nature of the study.

Electrolyte abnormalities were defined according to institutional laboratory reference ranges, with hyponatremia defined as serum sodium levels below 135 mmol/L, hypokalemia as potassium levels below 3.5 mmol/L, and hypophosphatemia as phosphate levels below 0.8 mmol/L.

Statistical analysis

Statistical analyses were performed using R software (version 4.5.1), with statistical significance defined as a two-sided p-value < 0.05. Continuous variables are reported as mean ± SD for normally distributed data and as median (IQR) for non-normally distributed data. Comparisons between patients with and without AKI were conducted using two-sample t-tests for normally distributed variables and Wilcoxon rank-sum tests for non-normally distributed variables. Categorical variables are presented as frequencies and percentages and were compared using Fisher’s exact test.

Associations between clinical variables and the risk of AKI were examined using logistic regression analysis, with results expressed as odds ratios and 95% confidence intervals. Time-to-event analyses were performed using Kaplan-Meier methods to evaluate hospitalization duration and outcomes following CAR-T-cell therapy. Cox proportional hazards regression models were used to estimate hazard ratios and corresponding 95% confidence intervals for factors associated with clinical outcomes.

## Results

Baseline characteristics

This study included 16 patients with hematological malignancies who underwent CAR-T-cell therapy (n = 8 (50%) male; mean age: 56.7 ± 17.7 years). Of the 16 patients, n = 6 (37.5%) had hypertension, n = 5 (31.3%) had diabetes mellitus, and n = 3 (18.8%) had CKD. The mean serum Cr level was 70.1 ± 27.4 μmol/L, and the mean estimated glomerular filtration rate (eGFR) was 96.9 ± 25.8 mL/min/1.73 m². All patients had previously undergone hematopoietic stem cell transplantation (HSCT) and were administered cyclophosphamide (500 mg/m²) and fludarabine (30 mg/m²). Regarding primary diagnosis, the most common was relapsed diffuse large B-cell lymphoma (DLBCL) (n = 7 (43.8%)), followed by refractory DLBCL (n = 3 (18.8%)) and other B-cell malignancies. Most patients (n = 12 (75.0%)) had received more than two prior lines of therapy. All patients developed electrolyte disturbances (n = 16 (100%)), with potassium disturbances being the most common (isolated: n = 3 (18.8%); with phosphate: n = 3 (18.8%)), followed by phosphate disturbance alone (n = 1 (6.3%)).

Prevalence of AKI and electrolyte disturbances

**Figure 1:**
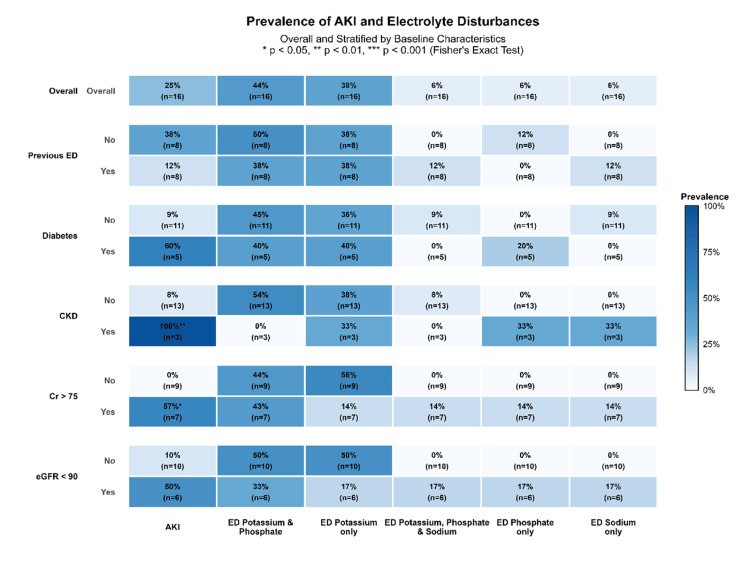
Overall and stratified prevalence heatmap of AKI and electrolyte disturbances after CAR-T therapy. AKI: Acute kidney injury; CAR-T: Chimeric antigen receptor-T-cell.

Figure 1 shows a heatmap of the prevalence rates of AKI and electrolyte disturbances after CAR-T-cell therapy. Of the 16 patients, 4 (25.0%) developed AKI, while all patients (n = 16 (100%)) developed electrolyte disturbances. Most patients developed potassium and phosphate co-disturbances (n = 7 (44.0%)), followed by potassium disturbance alone (n = 6 (38.0%)), sodium disturbance alone (n = 1 (6.0%)), phosphate disturbance alone (n = 1 (6.0%)), and potassium, phosphate, and sodium co-disturbance (n = 1 (6.0%)). Regarding baseline characteristics, all patients with pre-existing CKD developed AKI (n = 3 (100%), P < 0.01)

Risk factors for AKI

Table 1 presents the patients’ baseline characteristics by AKI status. Those who developed AKI were older (65.8 ± 9.9 vs. 53.7 ± 19.0 years, P = 0.251) and more often had diabetes mellitus (of 5 patients, n = 3 (75.0%) developed AKI vs. n = 2 (16.7%) who did not develop AKI, P = 0.051); however, these differences were not statistically significant, although the difference in the incidence of diabetes mellitus was borderline significant. Additionally, the prevalence of CKD (75.0% vs. 0.0%, p-value = 0.020) and mean serum Cr level (104.5 ± 19.4 vs. 58.7 ± 18.6 μmol/L, P < 0.001) were significantly higher in those with AKI than in those without AKI, while the mean eGFR was significantly lower (69.8 ± 18.4 vs. 106.0 ± 21.4 mL/min/1.73 m², P = 0.009). However, the prevalence of hypertension, primary diagnosis, prior lines of therapy, or electrolyte disturbances did not differ significantly between those with and without AKI (all P > 0.05).

**Table 1:** Baseline characteristics according to AKI status. Data are presented as mean (SD) and n (%) for continuous and categorical variables, respectively; two-sample t-tests and Fisher’s exact tests were used to obtain p-values. AKI: Acute kidney injury; eGFR: Estimated glomerular filtration rate; CAR-T: Chimeric antigen receptor T-cell; DLBCL: Diffuse large B-cell lymphoma; PMBCL: Primary mediastinal B-cell lymphoma; HCT: Hematopoietic cell transplantation; Cy: Cyclophosphamide; Fluda: Fludarabine.

Variable	Overall (N = 16)	No AKI (N = 12)	AKI (N = 4)	P-value
Age (years)	56.7 (17.7)	53.7 (19.0)	65.8 (9.9)	0.251
Sex				0.268
Female	8 (50.0%)	7 (58.3%)	1 (25.0%)	
Male	8 (50.0%)	5 (41.7%)	3 (75.0%)	
Hypertension				0.554
No	10 (62.5%)	8 (66.7%)	2 (50.0%)	
Yes	6 (37.5%)	4 (33.3%)	2 (50.0%)	
Diabetes mellitus				0.051
No	11 (68.8%)	10 (83.3%)	1 (25.0%)	
Yes	5 (31.3%)	2 (16.7%)	3 (75.0%)	
Chronic kidney disease				0.02
No	13 (81.3%)	12 (100.0%)	1 (25.0%)	
Yes	3 (18.8%)	0 (0.0%)	3 (75.0%)	
Serum creatinine (μmol/L)	70.1 (27.4)	58.7 (18.6)	104.5 (19.4)	<0.001
eGFR (mL/min/1.73 m²)	96.9 (25.8)	106.0 (21.4)	69.8 (18.4)	0.009
Indication for CAR-T-cell therapy (primary diagnosis)				0.9
DLBCL	4 (25.0%)	3 (25.0%)	1 (25.0%)	
PMBCL	1 (6.3%)	1 (8.3%)	0 (0.0%)	
Refractory DLBCL	3 (18.8%)	3 (25.0%)	0 (0.0%)	
Relapsed DLBCL	7 (43.8%)	4 (33.3%)	3 (75.0%)	
T-cell-rich B-cell lymphoma	1 (6.3%)	1 (8.3%)	0 (0.0%)	
Prior lines of therapy				0.203
Less than two	4 (25.0%)	2 (16.7%)	2 (50.0%)	
More than two	12 (75.0%)	10 (83.3%)	2 (50.0%)	
History of autologous or allogeneic HCT				NA
Yes	16 (100.0%)	12 (100.0%)	4 (100.0%)	
Lymphodepletion regimen				NA
Cy 500/Fluda 30	16 (100.0%)	12 (100.0%)	4 (100.0%)	
Electrolyte disturbances (pattern)				0.2
No	8 (50.0%)	5 (41.7%)	3 (75.0%)	
Yes - potassium and phosphate	3 (18.8%)	3 (25.0%)	0 (0.0%)	
Yes - phosphate	1 (6.3%)	1 (8.3%)	0 (0.0%)	
Yes - potassium	3 (18.8%)	3 (25.0%)	0 (0.0%)	
Yes - sodium	1 (6.3%)	0 (0.0%)	1 (25.0%)	

**Figure 2:**
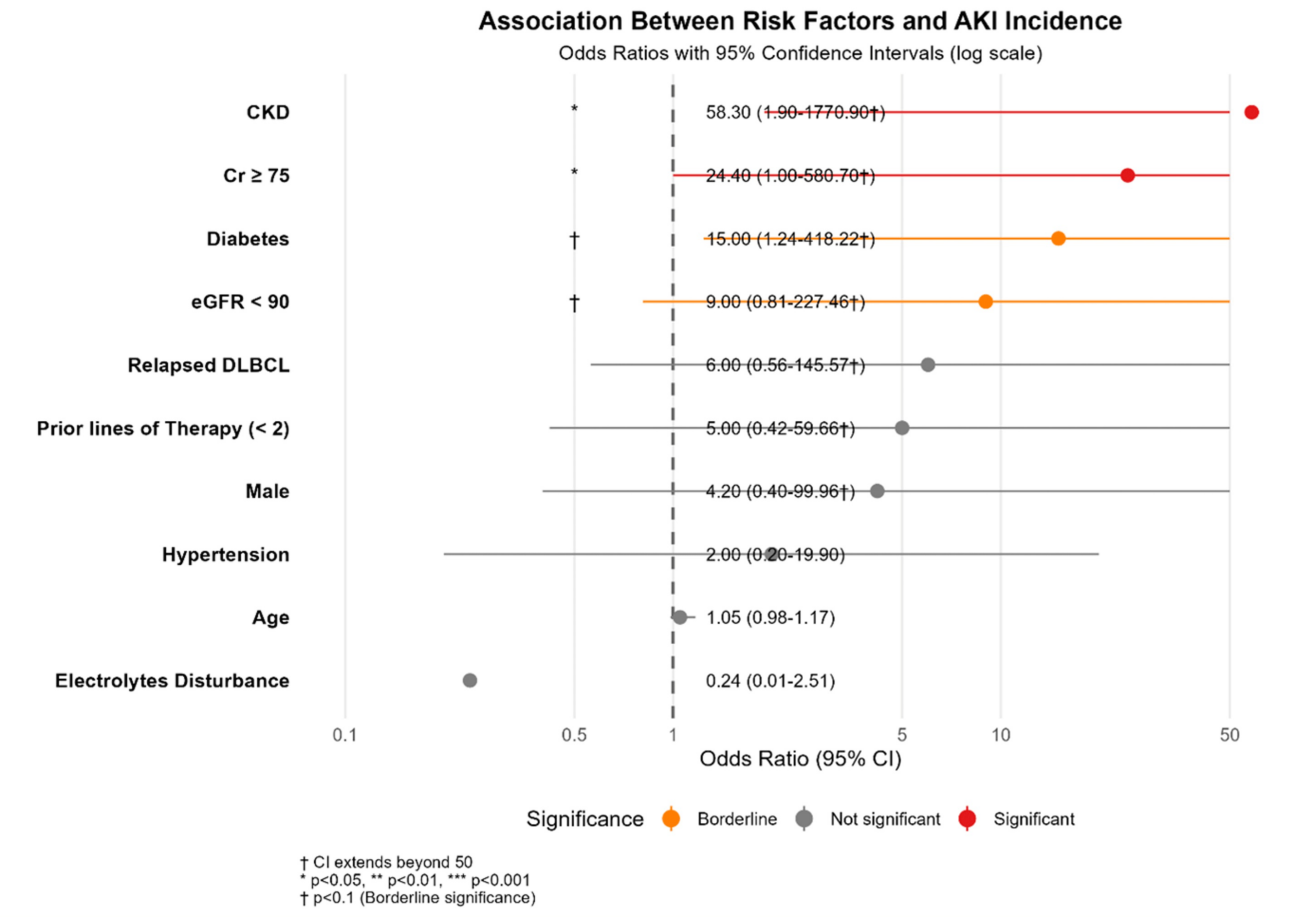
Odds of AKI according to potential risk factors. AKI: Acute kidney injury; CKD: Chronic kidney disease: Cr: Creatinine; eGFR: Estimated glomerular filtration rate; DLBCL: Diffuse large B-cell lymphoma.

Table 2 and Figure 2 present the impact of patients’ baseline characteristics on the risk of developing AKI. Pre-existing CKD was the strongest predictor of AKI (OR: 58.3, 95% CI: 1.9-1770.9, P = 0.020), followed by pre-existing diabetes mellitus (OR: 15.0, 95% CI: 1.24-418.22, P = 0.051). In contrast, male sex, hypertension, and prior lines of therapy (<2) showed non-significant trends toward an increased risk of AKI (all P > 0.05). Similarly, age, electrolyte disturbances, and relapsed DLBCL were not significantly associated with AKI (all P > 0.05).

**Table 2:** Risk factors for AKI. Data are presented as OR (95% CI); P values were obtained from logistic regression analysis. Statistical significance was set at P < 0.05. AKI: Acute kidney injury; CKD: Chronic kidney disease; Cr: Creatinine; eGFR: Estimated glomerular filtration rate; DLBCL: Diffuse large B-cell lymphoma.

Variable	OR (95% CI)	P-value
Age (years)	1.1 (1.0-1.2)	0.251
Male sex	4.2 (0.40-100.0)	0.268
Hypertension (yes)	2.0 (0.20-19.9)	0.554
Diabetes mellitus (yes)	15.0 (1.2-418.2)	0.051
Pre-existing CKD (yes)	58.3 (1.9-1770.9)	0.02
Serum creatinine ≥ 75 μmol/L (yes)	24.4 (1.0-580.7)	0.048
eGFR < 90 mL/min/1.73 m² (yes)	9.0 (0.8-227.5)	0.099
Relapsed DLBCL (yes)	6.0 (0.6-145.6)	0.17
Prior lines of therapy < 2	5.0 (0.4-59.6)	0.203
Electrolyte disturbance(s) (yes)	0.2 (0.0-2.5)	0.268

CAR-T-cell therapy outcomes by AKI status

Table 3 compares patients’ clinical outcomes by AKI status. All patients experienced CRS, and the incidence of immune effector cell-associated neurotoxicity syndrome (ICANS) (P = 0.554) and exposure to nephrotoxic agents (P = 0.644) did not differ significantly between those with and without AKI. All four patients who developed AKI were classified as stage 1. Notably, only one patient with AKI (25.0%) achieved renal recovery, with a recovery time of one day. ICU admission rates (n = 3 (75.0%) vs. n = 1 (25.0%); P = 0.099) and the median hospitalization duration (39 vs. 29 days, P = 0.301) were not significantly higher in patients with AKI compared with those without AKI. No cases of tumor lysis syndrome or early mortality were observed. The incidence of electrolyte disturbances did not differ significantly between patients with and without AKI (P = 0.264).

**Table 3:** Clinical outcomes by AKI status. Data are presented as median (quartile 1-quartile 3) for non-normally distributed continuous variables. P-values were obtained using the Wilcoxon rank-sum test for continuous variables and Fisher’s exact test for categorical variables. AKI: Acute kidney injury; CKD: Chronic kidney disease; CRS: Cytokine release syndrome; ICANS: Immune effector cell-associated neurotoxicity syndrome; Q1: First quartile; Q3: Third quartile.

Outcome	Overall (N = 16)	No AKI (N = 12)	AKI (N = 4)	P-value
Renal outcomes				
AKI stage				-
None	12 (75.0%)	12 (100.0%)	0 (0.0%)	
Stage 1	4 (25.0%)	0 (0.0%)	4 (100.0%)	
Renal recovered	1 (25.0%)	-	1 (25.0%)	-
Time to renal recovery (days)				-
Median (Q1-Q3)	1.0 (1.0-1.0)	-	1.0 (1.0-1.0)	
CKD	4 (25.0%)	1 (8.3%)	3 (75.0)	0.027
Electrolyte disturbance				0.264
Yes - potassium and phosphate	7 (43.8%)	6 (50.0%)	1 (25.0%)	
Yes - phosphate	1 (6.3%)	0 (0.0%)	1 (25.0%)	
Yes - potassium	6 (37.5%)	5 (42.0%)	1 (25.0%)	
Yes - sodium	1 (6.3%)	0 (0.0%)	1 (25.0%)	
Yes - potassium, phosphate, and sodium	1 (6.3%)	1 (8.3%)	0 (0.0%)	
Toxicity associations				
CRS at any stage	16 (100.0%)	12 (100.0%)	4 (100.0%)	-
ICANS				0.554
No	10 (62.5%)	8 (67.0%)	2 (50.0%)	
Yes	6 (37.5%)	4 (33.0%)	2 (50.0%)	
Tumor lysis syndrome				-
No	16 (100.0%)	12 (100.0%)	4 (100.0%)	
Nephrotoxic agents				0.644
None	2 (12.5%)	2 (17.0%)	0 (0.0%)	
Vancomycin	14 (87.5%)	10 (83.0%)	4 (100.0%)	
Hospitalization, ICU metric and mortality				
ICU admission				0.099
No	10 (62.5%)	9 (75.0%)	1 (25.0%)	
Yes	6 (37.5%)	3 (25.0%)	3 (75.0%)	
Early mortality (30 days)				-
No	16 (100.0%)	12 (100.0%)	4 (100.0%)	
Length of hospitalization (days)				0.301
Median (Q1, Q3)	29.0 (25.5, 33.0)	29.0 (25.0, 32.5)	39.0 (27.0, 52.5)	

**Figure 3:**
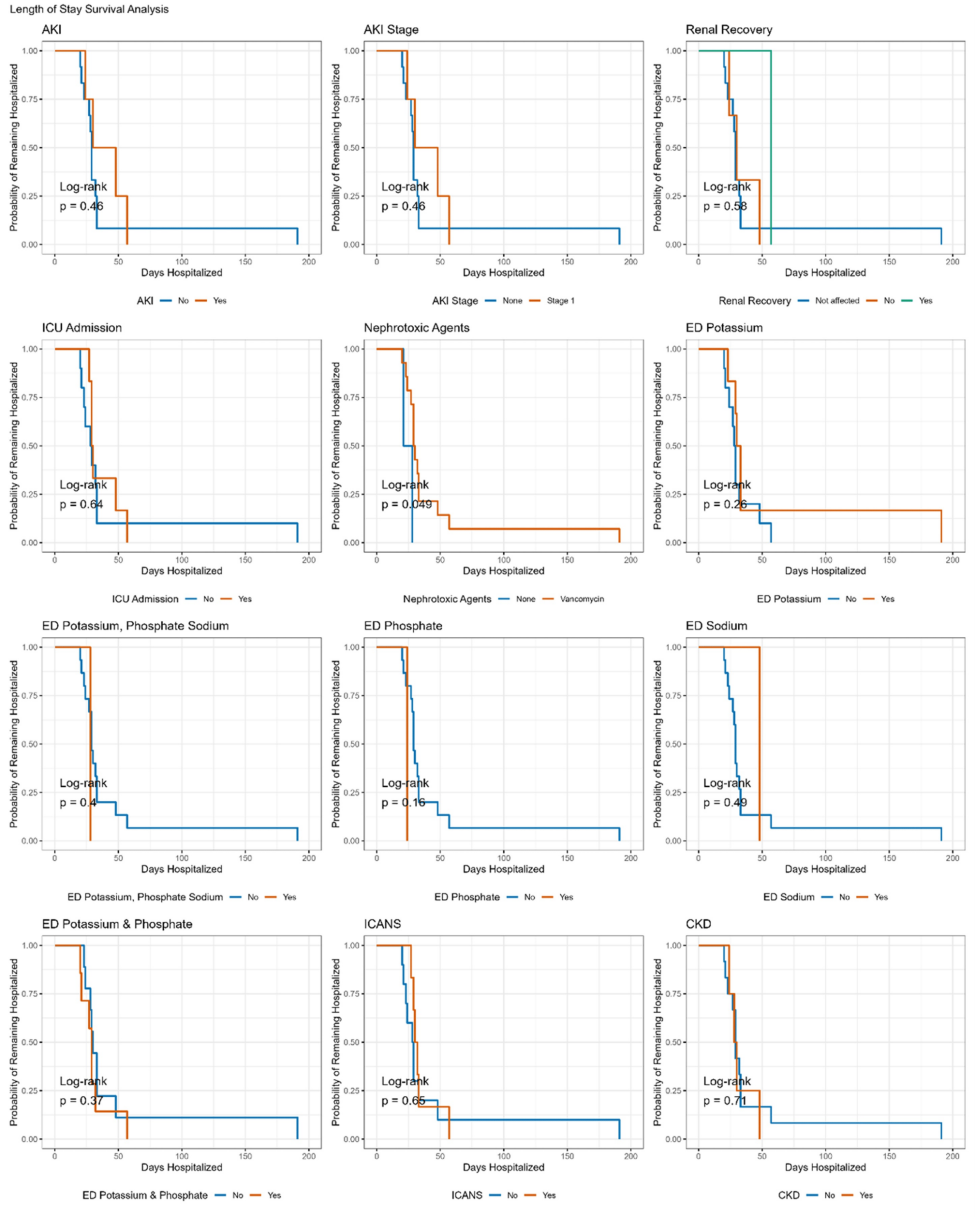
Length of stay according to CAR-T outcomes in all patients. CAR-T: Chimeric antigen receptor T-cell.

**Figure 4:**
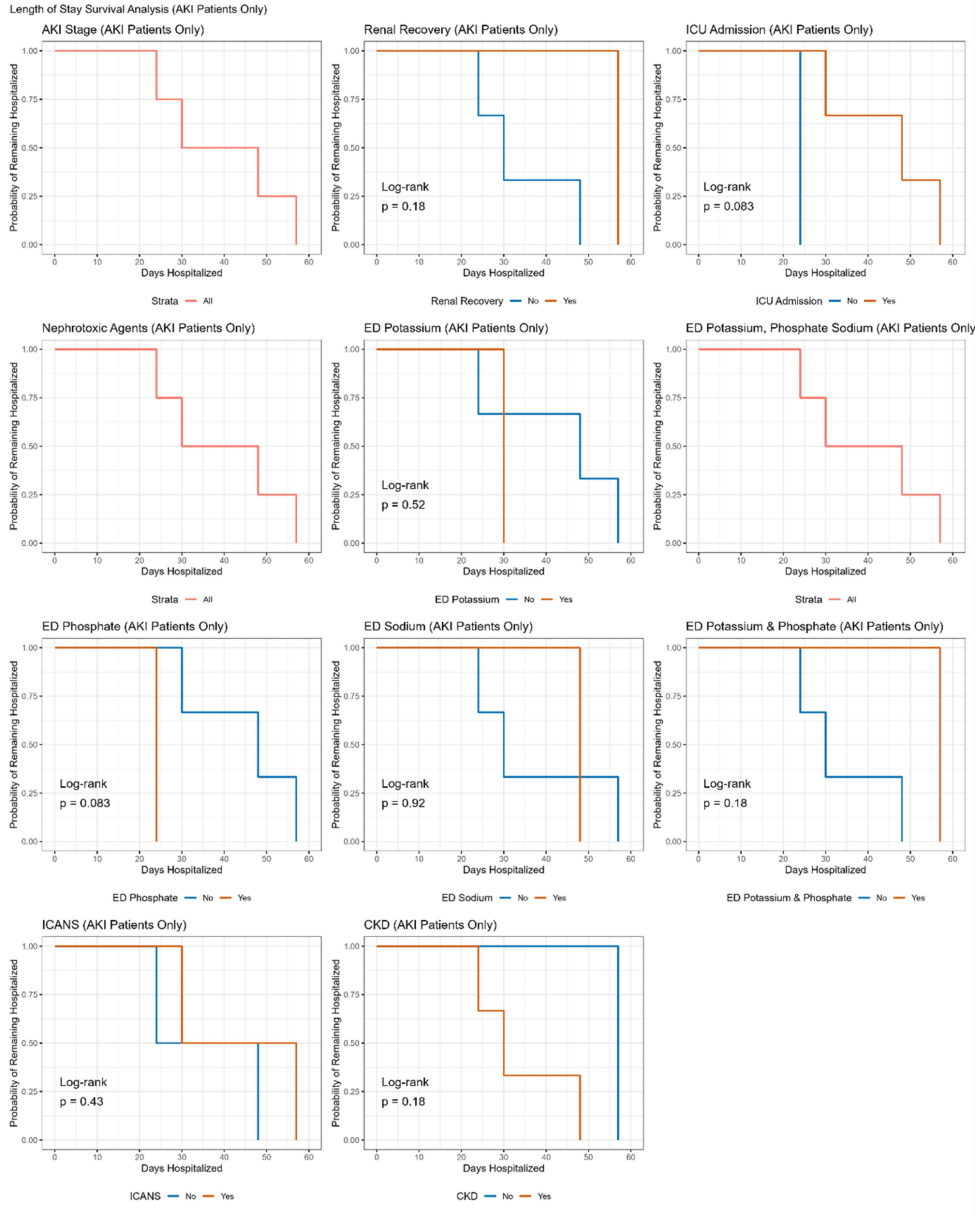
Length of stay according to CAR-T outcomes in patients with AKI. CAR-T: Chimeric antigen receptor T-cell; AKI: Acute kidney injury.

Kaplan-Meier survival analysis revealed that hospitalization duration was significantly predicted only by exposure to nephrotoxic agents (P = 0.049, log-rank test), while the remaining clinical outcomes after CAR-T-cell therapy did not significantly predict hospitalization duration (all P > 0.05). Subsequent analysis using a Cox regression model revealed a marginally significant association (HR: 0.21, 95% CI: 0.04-1.17, P = 0.074), indicating a 79% lower risk of extended hospitalization among patients not administered vancomycin. As shown in Figures 3-4, none of the outcomes significantly predicted hospitalization duration in patients with AKI (all P > 0.05, log-rank test).

## Discussion

In our retrospective cohort, the incidence of AKI did not deviate from the previously reported range, including in Farooqui N et al., where n = 14 (17.0%) of the 83 included patients developed AKI (stage 1: n = 11 (13.0%)) [15]; León-Román J et al., where n = 17 (14.8%) of the 115 included patients developed stage 1 AKI [19]; and Wood AC et al., where n = 38 (23%) of the 166 included patients developed AKI [20].

The risk factors for AKI identified in our study may help reduce the incidence of AKI among patients receiving CAR-T-cell therapy. In our cohort, CKD was the strongest predictor of AKI (OR: 58.3, 95% CI: 1.9-1770.9, P = 0.020), followed by diabetes mellitus (OR: 15.0, 95% CI: 1.24-418.22, P = 0.051). In addition, male sex, hypertension, and fewer prior lines of therapy (<2) showed non-significant trends toward an increased risk of AKI (all P > 0.05). However, age, electrolyte disturbances, and relapsed DLBCL did not significantly influence the risk of AKI (all P > 0.05).

León-Román J et al. identified pre-existing CKD (adjusted OR: 8.20, 95% CI: 1.52-44.3, P = 0.02) and male sex (adjusted OR: 6.54, 95% CI: 1.14-37.6, P = 0.04) as significant risk factors for AKI [19]. Similar findings have also been reported by Ahmed G et al. [21].

In our study, we sought to identify the more common electrolyte disturbances. Interestingly, we observed hyponatremia (serum sodium <135 mmol/L) in n = 1 (6%) of our patients, which is markedly lower than the prevalence of n = 59 (75%) reported by Gupta S et al. among 78 patients [17]. In our cohort, we also observed that n = 6 (38.0%) had a potassium disturbance (serum potassium <3.5 mmol/L), n = 7 (44.0%) had potassium and phosphate co-disturbance, and n = 1 (6.0%) had a phosphate disturbance. A prospective study in Spain including 63 patients reported an incidence of n = 49 (78%) for hypophosphatemia, n = 44 (70%) for hypokalemia, and n = 37 (59%) for hyponatremia [19].

In the prospective cohort reported by Moncho-Francés F et al. [22], electrolyte abnormalities were observed in 60 (95%) of 63 patients, with hypophosphatemia emerging as the predominant disturbance.

The causes of electrolyte disturbances after CAR-T-cell therapy have not yet been fully elucidated, and these abnormalities may resolve spontaneously or require minimal intervention.

Finally, we did not find AKI to be significantly associated with longer hospitalization, ICU admission, or early mortality (Figures 3-4). Similarly, Gutgarts V et al. [23] reported no significant association with mortality in patients with AKI. In contrast, Russo E et al. [24] found AKI to be significantly associated with longer hospitalization and early mortality, and Vincendeau M et al. [25] reported AKI as an independent risk factor for in-hospital mortality.

Limitations

The results should be interpreted with caution in light of several methodological aspects. Data were obtained retrospectively from a single institution and involved a limited number of patients, which may restrict broader applicability. Urine output measurements were not consistently documented and therefore could not be incorporated into the definition of AKI. In addition, the regression and time-to-event analyses were intended to explore associations rather than provide definitive causal inferences, and confirmation of these findings in larger, multicenter populations is required.

## Conclusions

In this patient cohort, renal complications observed following CAR-T-cell therapy were predominantly mild in severity. The presence of underlying CKD and diabetes mellitus was associated with a higher likelihood of developing AKI. These findings underscore the importance of careful surveillance of renal function throughout therapy, along with heightened attention to modifiable risk factors such as exposure to nephrotoxic agents, contrast administration, and volume depletion. A more comprehensive understanding of renal injury mechanisms and their broader clinical implications will require future studies involving larger patient populations and longer durations of follow-up.
